# Clinical, Diagnostic, and Metabolic Characteristics Associated with Nephrolithiasis in the Black Women’s Health Study

**DOI:** 10.3390/jcm13195948

**Published:** 2024-10-07

**Authors:** Maria D’Amico, Richard K. Babayan, David S. Wang, Shaun Wason, Yvette C. Cozier

**Affiliations:** 1 Department of Urology, Thomas Jefferson University Hospital, Philadelphia, PA 19107, USA; mariajdamico3@gmail.com; 2 Department of Urology, Boston University/Boston Medical Center, 725 Albany St. Suite 3B, Boston, MA 02118, USA; rbabayan@bu.edu (R.K.B.); wangdsw@bu.edu (D.S.W.); 3 Division of Urology, Beth Israel Deaconess Medical Center, 330 Brookline Ave, Boston, MA 02215, USA; swason@bidmc.harvard.edu; 4 Slone Epidemiology Center at Boston University, Boston University Chobanian & Avedesian School of Medicine, 72 East Concord Street, L-7, Boston, MA 02118, USA; 5 Department of Epidemiology, Boston University School of Public Health, 715 Albany Street, Talbot-3E, Boston, MA 02118, USA

**Keywords:** nephrolithiasis, kidney stones, black, women, African American, metabolic evaluation

## Abstract

**Background/Objectives:** Nephrolithiasis (kidney stones) is a frequently occurring urologic condition resulting in significant patient morbidity and healthcare costs. Despite the higher prevalence of metabolic risk factors for nephrolithiasis among Black women, there have been few epidemiologic studies of kidney stones focusing on this group. **Methods:** We describe demographic and health characteristics, diagnostics, and metabolic profiles of US Black women with self-reported kidney stones. The women were participants in the Black Women’s Health Study (BWHS), a large prospective cohort of US Black women (median age 38 years) begun in 1995. **Results:** Among the 2750 BWHS participants who completed an online supplemental questionnaire assessing urologic health, 201 women reported nephrolithiasis. Of this number, 62% had completed ≥ 16 years of education, and 82% reported access to health care. Overall, 39% reported experiencing ≥ 2 stones in their lifetime, and 29% required surgery to treat the condition. Thirty-two percent reported having completed a metabolic evaluation, while 70% had undergone a CT scan to diagnose nephrolithiasis. The frequency of metabolic evaluation increased with the number of metabolic components reported: 3% (0 components) to 43% (3–4 components). **Conclusions:** Our findings are consistent with reports of lower rates of metabolic evaluation among Black patients despite their having multiple risk factors for nephrolithiasis. Further study is needed to identify the barriers and facilitators of metabolic and diagnostic workup of nephrolithiasis in Black women.

## 1. Introduction

Nephrolithiasis, commonly referred to as kidney stones, is a frequently occurring urologic condition resulting in significant patient morbidity and healthcare costs [1,2]. In the United States, approximately 240 out of every 100,000 people are estimated to develop kidney stones annually [3]. Treatment options depend upon the size and location of the stone. For example, small stones may pass with increased hydration, while larger stones may require surgical intervention [4]. Studies suggest that up to 50% of patients experience recurrent disease, highlighting the importance of ongoing management and prevention [5].

Kidney stones can result from genetic, dietary, and environmental factors and form when certain substances in the urine, such as calcium, oxalate, uric acid, or cystine, crystallize and aggregate [6]. Dehydration increases the concentration of stone-forming substances in the urine [7]. Thus, the global incidence of nephrolithiasis varies according to geographic location; higher prevalence has been observed in areas with warmer climates where dehydration can occur more frequently [8]. Dietary factors, such as high sodium, excessive animal protein, and oxalate-rich foods, contribute to stone formation [7]. Metabolic conditions including hypercalciuria and hyperuricosuria also increase risk; additionally, conditions such as obesity, type 2 diabetes, and hypertension are associated with stone formation [9,10,11].

The risk of nephrolithiasis is estimated to be higher in men compared to women (10.6% versus 7.1%, respectively) and more commonly reported among White (10.3%) than Black patients (4.3%) [12]. However, the prevalence of the condition has been increasing, especially among Black women [3,13] who have higher rates of components of the metabolic syndromes (e.g., obesity, type 2 diabetes) [9,14,15] that have been linked to kidney stone formation [16,17]. For example, a recent study in a cohort of US Black women found positive associations between metabolic and dietary factors, gallstones, and the risk of kidney stone formation [17].

Clinical metabolic evaluation, including blood analysis and 24-hour urine collection, is a key tool for predicting risk and lowering the chances of recurring kidney stones [18]. Despite their high levels of risk factors for kidney stone disease, no studies have exclusively analyzed the clinical and metabolic characteristics of black women. We report on data collected in the Black Women’s Health Study (BWHS) from a subset of participants with a history of nephrolithiasis regarding diagnosis and treatments for stones, including risk factors associated with completing a metabolic workup within this population.

## 2. Materials and Methods

### 2.1. The Black Women’s Health Study (BWHS)

The BWHS was begun in 1995 with the goal of addressing the dearth of studies focused on understanding observed health disparities among African-American/Black women for numerous conditions, including cancer and cardiovascular disease [19]. A convenience sample of over 64,000 Black women aged 21–69 years (median age: 38 years) from across the United States enrolled by completing a 14-page postal health questionnaire [19,20,21]. Participants were primarily subscribers to Essence magazine (Essence.com (Accessed on 5 October 2024), a publication targeted to African-American/Black women and widely circulated in the African-American/Black Community since 1970 [19]. Members of African-American/Black professional groups were also targeted for enrollment, including the National Black Nurses Association (nbna.org (Accessed on 5 October 2024) and the National Education Association (NEA) Black Caucus (neablackcaucus.org (Accessed on 5 October 2024) [19,21]. Additionally, a snowball approach to recruitment [22] was applied by asking early responders to the questionnaire to nominate family, friends, and associates who might be interested in participating in a longitudinal study [21]. Those nominated were then mailed a questionnaire and given the same opportunity to recommend others.

The first follow-up questionnaire was mailed in 1997 and asked about the participant’s race/ethnicity; 99.6% of respondents self-identified as African-American or Black. Women who did not identify as African-American or Black were excluded. Thus, the 59,000 women with valid addresses comprise the BWHS cohort, which has been followed to the present [21]. Beginning with the 2003 follow-up cycle, all BWHS participants were extended the option of completing either a mailed paper version or an online version of the study questionnaire [23].

Study participants represent all regions of the United States, with over 80% residing in California, the District of Columbia, Georgia, Illinois, Indiana, Louisiana, Maryland, Massachusetts, Michigan, New York, New Jersey, South Carolina, and Virginia [20,21]. Biennial follow-up through postal or online questionnaires has been successful for >85% of potential person-years of follow-up after 13 questionnaire cycles (through 2021).

The 1995 baseline questionnaire collected information on a wide range of variables, including demographic factors, reproductive, behavioral, anthropometric, existing medical conditions, and medications. Follow-up questionnaires, starting in 1997, ascertained newly occurring (incident) health conditions and updated information on weight, physical activity, smoking, alcohol use, parity, menopause, and other factors. The Institutional Review Board (IRB) of Boston University School of Medicine approved the study protocol. Participants indicated consent by completing and returning study questionnaires.

### 2.2. Supplemental Urinary Tract Function Questionnaire

In 2017, a subset of BWHS participants were invited to complete an online self-administered supplemental questionnaire aimed at collecting data on urinary tract function, including urinary incontinence (UI), urinary tract infection, and kidney stones. We emailed invitations and a link to the supplemental questionnaire to a random sample of 5780 participants who had previously completed the online version of the 2011, 2013, and 2017 follow-up BWHS questionnaires, and who had indicated in either the 2011 or 2013 questionnaire that they had monthly or more frequent experiences of UI. The supplemental questionnaire included the following questions on kidney stones: “Have you ever been diagnosed with kidney stones?” (yes, no); If yes, “How many kidney stones in the last year?” (number); ”How many kidney stones in your lifetime?” (number); “Age at first kidney stone?” (years); “Do you currently have kidney stones?” (yes, no); If yes, “How many CT scans for kidney stones?” (number); “How many surgical procedures for kidney stones?” (number); and “Have you ever had a metabolic workup (24-hour urine collection) to determine your risk for developing a kidney stone?” (yes, no).

### 2.3. Covariates

Data on years of completed education (≤12, 13–15, ≥16) was collected at baseline (1995) and on the 2003 follow-up questionnaires. Data on height (feet and inches) was collected at baseline. Data on age (years), current weight (pounds), cigarette smoking (ever, never), alcohol consumption (<1, 1–6, ≥7 drinks/week), history of type-2 diabetes (yes, no), hypertension (yes, no), and hyperlipidemia (high cholesterol level) (yes, no), and whether the participant had a recent medical visit (yes, no) were collected on most follow-up questionnaires. Body Mass Index (BMI) was calculated as weight (converted to kilograms), divided by height (converted to square meters) (kg/m^2^), and divided into categories representing “normal” (<25 kg/m^2^), “overweight” (25–29 kg/m^2^), and “obese” (≥30 kg/m^2^) [24]. The values used in this report were ascertained on the 2017 questionnaire.

### 2.4. Data Analysis

The current descriptive analysis is based on the 201 women who, on the supplemental questionnaire, reported a history of nephrolithiasis and answered questions regarding testing and treatment for kidney stones in 2017. We calculated means, standard deviations, and proportions (chi-square tests) to summarize sample characteristics.

## 3. Results

Table 1 presents data on the characteristics of the BWHS participants who were invited to complete the supplemental questionnaire (n = 5780) and those who completed the questionnaire (n = 2750). The women who completed the supplemental questionnaire were similar to the larger group of BWHS participants invited to complete the UI web questionnaire in terms of age (mean: 59.3 (SD: 8.6), years vs. mean: 59.1 (SD: 8.8), years), BMI (mean: 31.9 (SD: 7.2), kg/m^2^ for both groups), highest educational attainment (≥16 years: 57% vs. 56%), region of the US in which they lived (Northeast: 28% vs. 27%; South: 39% vs. 41%; Midwest: 19% for both groups; and West: 14% vs. 13%), smoking (37% vs. 35%), alcohol consumption (≥1 drink per week: 46% vs. 45%), having a recent medical visit (79% vs. 78%), and prevalence of type 2 diabetes (21% vs. 23%), hypertension (59% for both groups), and hyperlipidemia (52% vs. 51%). The women who reported a history of nephrolithiasis (n = 201) were also similar to the supplemental questionnaire group in terms of age (mean: 32.6 (SD: 7.1), years vs. mean: 31.9 (SD: 7.2), years), BMI (mean: 60.8 (SD: 8.6) vs. mean: 59.3 (SD: 8.6), kg/m^2^), smoking (38% vs. 37%), and geographic region. They were, however, slightly more educated than the population from which they were drawn (≥16 years of education: 62% vs. 57%) and were more likely to have visited a doctor recently (82% vs. 79%) but were less likely to consume one or more weekly alcoholic beverages (38% vs. 46%). They also had higher prevalence of hypertension (71% vs. 59%), hyperlipidemia (58% vs. 51%), and type-2 diabetes (31% vs. 23%); they were also more likely to concurrently report three or more metabolic syndrome traits (40% vs. 29%).

Table 2 presents the clinical characteristics of nephrolithiasis reported by sub-study participants. On average, participants experienced their first kidney stone at age 45 (range: 16–82 years), and 19% had the condition at the time of the questionnaire. Furthermore, 39% experienced > 2 stones in their lifetime, with 26% reporting at least one occurrence in the last year. Approximately one third (32%) had completed a metabolic workup for their kidney stones, while 70% had undergone a CT scan, and 29% required a surgical procedure to treat their nephrolithiasis. Among those who completed a metabolic workup, more than half had a BMI over 30 kg/m^2^, and 38% had type-2 diabetes.

In Table 3, we describe the frequency of metabolic workup according to the clinical characteristics of the BWHS sub-study participants who completed a metabolic evaluation. Those evaluated were more likely to be aged 40 years and older (69%), to have experienced >1 stone in their lifetime (57%), and to have undergone one or more CT scans (89%) or surgery (54%). The frequency of evaluation also increased as the number of metabolic syndrome components (obesity, type-2 diabetes, hypertension, hyperlipidemia) increased. For example, those with no components were the least likely to report undergoing a metabolic workup (3%); this number increased to 22% for those reporting one component, to 32% for those reporting two components, and 43% for those reporting three to four components.

## 4. Discussion

In this study, we report data from a subset of participants with a history of nephrolithiasis in the Black Women’s Health Study, a prospective epidemiologic follow-up of US black women. Among the sample of women who had kidney stones, there was a high prevalence of three or more concomitant metabolic syndrome traits. Additionally, there were high reports of stone recurrence and use of CT scans to evaluate kidney stones, but overall low rates of metabolic evaluation of risk. In particular, 39% reported having experienced two or more stones in their lifetime. This recurrence rate is consistent with previous reports of 30–40% [5,25], and highlights the importance of secondary prevention in urinary stone disease. A metabolic evaluation can assist in directing preventive measures [26]. However, only 32% of study participants reported completing a metabolic workup. Metabolic evaluation was highest among women reporting recurrent stones (57%). The European (EUA) and American Urological Association (AUA) each recommend that metabolic workups be performed in interested first-time and recurrent stone formers, or those considered high risk, such as those with obesity and type 2 diabetes mellitus [27,28,29]. In the present study, 58% of women reporting kidney stones were obese, and 31% reported a diagnosis of T2DM. However, only 54% and 38% of women with these conditions, respectively, reported undergoing a metabolic workup.

Few studies have analyzed the factors associated with completing a metabolic workup for kidney stones. Sninsky et al. found that low education, high poverty, and younger age are associated with lower rates of 24-h urine collection [30]. Ghiraldi et al. found that African-American/Black patients were nearly half as likely to submit a 24-h urine sample compared to White/Caucasian patients (30.9% vs. 51.8%; *p* < 0.05), while patients with a family history of stones were twice as likely to submit a urine sample compared to those without [31]. In our study, in which participants are educated, older, and reported a medical visit within the previous 2-year period, a low proportion of women reported submitting a 24-h urine sample. Thus, age, education, and poverty alone do not account for the low frequency of metabolic testing. Other possible contributors to low metabolic evaluation include higher levels of medical mistrust or poorer communication between patients and providers, in addition to the inconvenience of sample collection [32]. We did not collect information on those factors.

There is also evidence that provider practice patterns strongly influence the use of metabolic evaluation for stone disease. An analysis of medical claims data found that the specialty of the follow-up provider after an acute stone event influences whether or not a metabolic evaluation is performed [33]. A survey conducted among North American members of the Endourological Society found that only 5% of respondents reported collecting 24-h samples in all first-time stone formers, and only 6% collected samples in all clinical scenarios. Furthermore, less than half reported collecting samples in all recurrent stone formers, and only a third collected samples in high-risk first-time stone formers [34]. Further research is needed to assess barriers to compliance in both patients and providers, as results from this testing can lead to future stone prevention.

CT scans are widely recognized as the preferred imaging method for diagnosing kidney stones, and their usage has notably been on the rise [31,35]. In our study, 70% of participants diagnosed with a kidney stone reported undergoing a CT scan, aligning with the current literature. Due to the cross-sectional nature of our data, however, we could not determine whether CT scans were used to diagnose recurrent stone formation. Analyzing data from both the National Hospital Ambulatory Medical Care Survey and the National Health and Nutrition Examination Survey, Fwu and colleagues observed an increase in CT scan utilization for urolithiasis, from 21% to 71% between 1998/2000 and 2007/2009 [36]. Although CT scans are highly effective in detecting stones, their drawbacks include cost and radiation exposure [35]. For patients with recurrent stones, this can lead to significant radiation exposure [37] and future health risks [38]. Reducing kidney stone formation through metabolic evaluation can effectively decrease radiation exposure and associated health risks for patients.

Twenty-nine percent of our sample reported undergoing surgery for their stones, of which 5% underwent three or more procedures. Results from prior studies have reported frequencies between 20–38% of stone formers undergoing surgery [39,40], with approximately 7% undergoing repeat surgical procedures [40]. Among the possible reasons for surgery is the significant economic impact on work productivity associated with kidney stones, which may prompt patients to choose treatment rather than waiting for the stone to pass spontaneously [41]. Additionally, first-time stone formers with narrower ureters may face greater symptoms and difficulties in stone passage, leading them to opt for surgical intervention [40]. In a study by Portis et al., 38% of stone formers presenting to an emergency room within a major US metropolitan health system went directly to surgery. Of this group, symptoms were the primary reason for the procedure for 52% [40]. In the current analysis, we did not collect information on the indications for surgery.

Our study has notable strengths and limitations. The current analysis was limited by the small sample size. In addition, our data involved self-reported kidney stones without confirmation by medical record. In the Health Professions Follow-up Study, 97% of self-reported cases of kidney stones were confirmed by medical record review [42]. Validation studies of other conditions in the BWHS (e.g., sarcoidosis, type-2 diabetes, hypertension) have shown high degrees of accuracy of self-report [43,44,45]. While the possibility that the under-diagnosis of kidney stones may have introduced some degree of bias cannot be ruled out, the fact that approximately 80% of the analytic sample reported a recent medical visit suggests that any such bias is small. Furthermore, at baseline, 96% and 96.6% of all BWHS participants, respectively, reported seeing a healthcare provider and having their blood pressure checked [20]. The BWHS participants are not a random sample of US black women, and the study population underrepresents the 15% of Black women nationally of similar ages who did not complete high school [46]. However, the participants represent all areas of the United States [21]. Finally, the study sample used as the basis of the current analysis consisted entirely of women reporting urinary incontinence, which could have biased the results. For example, the lifetime prevalence of stones previously reported for black patients was 4.3%^3^, compared to 7.3% reported in the current study; a difference also possibly magnified by our limited sample size [17]. We did not query study participants about family history of nephrolithiasis. In addition, there has been a dearth of studies involving Black patients and kidney stones; thus, the true burden of nephrolithiasis in this population is not clear. Nevertheless, the overall number of participants reporting a metabolic workup (32%) was still lower than anticipated. When asking participants about undergoing a metabolic workup, we only asked one question (24-h urine collection). We did not specify other types of metabolic workup, such as blood levels, uric acid, or vitamin D. Thus, we may have underestimated the number of women undergoing evaluation for stones. The study’s strengths include data on important clinical characteristics, as well as the ongoing nature of the BWHS cohort. The similarity of the characteristics of women who answered the questionnaire on which the kidney stone data were reported to the random sample of BWHS participants from which that group was drawn suggests that the answers were an unbiased representation of characteristics of the larger cohort.

## 5. Conclusions

In summary, within this descriptive study of US Black women, among those with a history of kidney stones, we found a high percentage of metabolic conditions, high exposure to CT imaging, and low rates of metabolic evaluation. Given the high prevalence of metabolic risk factors for nephrolithiasis in Black women, more work is needed to close this gap in clinical practice.

## Figures and Tables

**Table 1:** Characteristics of BWHS sub-study invitees and participants, 2017.

	BWHS Participants Invited to Complete the UI Web Questionnaire (n = 5780)	BWHS Participants Who Completed the UI Web Questionnaire (n = 2570)	Sub-Study Participants with Kidney Stones (n = 201)
Age, years (mean)(SD)	59.1 (8.8)	59.3 (8.6)	60.8 (8.6)
Body Mass Index (kg/m^2^) (mean)(SD)	31.9 (7.2)	31.9 (7.2)	32.6 (7.1)
	%		
Body Mass Index (kg/m^2^)			
<25 (normal)	16	16	11
25–29 (overweight)	30	30	31
≥30 (obese)	54	54	58
Education (years)			
≤12	10	11	10
13–15	34	33	28
≥16	56	57	62
Geographic region			
Northeast	27	28	28
South	41	39	40
Midwest	19	19	20
West	13	14	13
Ever smoker	35	37	38
Alcoholic beverages/week ^1^			
<1	55	54	58
≥1	45	46	38
Missing	---	---	4
Recent Medical visit (yes)	78	79	82
Type-2 Diabetes	23	21	31
Hypertension	59	59	71
Hyperlipidemia	51	52	58

^1^ A total of eight (8) participants reporting kidneystones did not have data on weekly alcohol consumption in 2017.

**Table 2:** Clinical characteristics of BWHS sub-study participants reporting nephrolithiasis (n = 201), 2017.

Clinical Characteristic	%
Age at first kidney stone (years)	
Median (IQR)	45.4 (22.0)
Range	16–82
Number of stones in last year	
0	74
1	20
≥2	6
Number of stones in lifetime	
1	61
2	23
≥3	16
Currently have stones	19
Number of CT scans for stones	
0	29
1–2	59
≥3	11
Number of surgical procedures for stones	
0	71
1–2	24
≥3	5
Ever metabolic workup for stones (yes)	32
Among women with BMI ≥ 30 kg/m^2^	54
Among women with Type-2 diabetes	38

Percentages may not total 100 due to missing data (two subjects did not provide information on the number of CT scans).

**Table 3:** Frequency of metabolic workup according to clinical characteristics of BWHS sub-study participants (n = 64), 2017.

Clinical Characteristic	Ever Metabolic Work Up
	%
Age at first kidney stone (years)	
<30	15
30–39	16
40–49	25
50–59	21
≥60	23
Number of stones in lifetime	
1	43
2	27
≥3	30
Number of CT scans for stones	
0	11
1–2	67
≥3	22
Number of surgical procedures for stones	
0	46
1–2	38
≥3	16
Number of metabolic syndrome components ^†^	
0	3
1	22
2	32
3–4	43

^†^ Metabolic syndrome components: obesity, type-2 diabetes, hypertension, and hypercholesterolemia.

## Data Availability

Data underlying the study cannot be made publicly available due to ethical concerns about patient confidentiality. Data will be made available to qualified researchers on request to BWHS@bu.edu.
